# Central Carbon Metabolism, Sodium-Motive Electron Transfer, and Ammonium Formation by the Vaginal Pathogen *Prevotella bivia*

**DOI:** 10.3390/ijms222111925

**Published:** 2021-11-03

**Authors:** Lena Schleicher, Sebastian Herdan, Günter Fritz, Andrej Trautmann, Jana Seifert, Julia Steuber

**Affiliations:** 1 Institute of Biology, University of Hohenheim, Garbenstraße 30, 70599 Stuttgart, Germany; lena.schleicher@uni-hohenheim.de (L.S.); sebastian.herdan@uni-hohenheim.de (S.H.); guenter.fritz@uni-hohenheim.de (G.F.); 2 HoLMiR-Hohenheim Center for Livestock Microbiome Research, University of Hohenheim, Leonore-Blosser-Reisen-Weg 3, 70599 Stuttgart, Germany; andrej.trautmann@uni-hohenheim.de (A.T.); seifert.jana@uni-hohenheim.de (J.S.); 3 Institute of Animal Science, University of Hohenheim, Emil-Wolff-Straße 8, 70599 Stuttgart, Germany

**Keywords:** bacterial vaginosis, *Prevotella bivia*, Na^+^-translocating NADH:quinone oxidoreductase, fumarate reductase, amino acid degradation

## Abstract

Replacement of the *Lactobacillus* dominated vaginal microbiome by a mixed bacterial population including *Prevotella bivia* is associated with bacterial vaginosis (BV). To understand the impact of *P. bivia* on this microbiome, its growth requirements and mode of energy production were studied. Anoxic growth with glucose depended on CO_2_ and resulted in succinate formation, indicating phosphoenolpyruvate carboxylation and fumarate reduction as critical steps. The reductive branch of fermentation relied on two highly active, membrane-bound enzymes, namely the quinol:fumarate reductase (QFR) and Na^+^-translocating NADH:quinone oxidoreductase (NQR). Both enzymes were characterized by activity measurements, in-gel fluorography, and VIS difference spectroscopy, and the Na^+^-dependent build-up of a transmembrane voltage was demonstrated. NQR is a potential drug target for BV treatment since it is neither found in humans nor in *Lactobacillus*. In *P. bivia*, the highly active enzymes L-asparaginase and aspartate ammonia lyase catalyze the conversion of asparagine to the electron acceptor fumarate. However, the by-product ammonium is highly toxic. It has been proposed that *P. bivia* depends on ammonium-utilizing *Gardnerella vaginalis*, another typical pathogen associated with BV, and provides key nutrients to it. The product pattern of *P. bivia* growing on glucose in the presence of mixed amino acids substantiates this notion.

## 1. Introduction

The most commonly reported microbiological syndrome among women in reproductive age is bacterial vaginosis (BV) [1]. This infection is associated with a variety of health issues, such as increased susceptibility to sexual transmitted pathogens, higher risk of pelvic inflammatory disease, or preterm births [1]. BV is characterized by a drastic change of the vaginal microbiome [2]. A healthy vagina is dominated by Gram-positive *Lactobacillus*, which maintain a vaginal pH of ~4.5 due to the degradation of sugars to lactic acid [3,4]. In BV, the vaginal microbiota is dominated by opportunistic pathogens such as *Gardnerella vaginalis* (earlier *Haemophilus vaginalis*), *Prevotella bivia*, or *Peptostreptococcus anaerobuis* [3,5,6].

*P. bivia* is a Gram-negative obligate anaerobic bacterium which, together with other *Prevotella* sp., accounts for up to 44% of bacterial species identified in BV patients [7]. It has the ability to invade the human cervix [8] and cause intrauterine infections [9]. *G. vaginalis* is another marker strain for BV and both *G. vaginalis* [10] and *P. bivia* [11] trigger BV phenotypes in mice models. It was proposed that amino acids released by *G. vaginalis* are metabolized by *P. bivia*, leading to a rise in ammonium concentration in a biofilm established by *G. vaginalis* and *P. bivia*. This increases the pH and might promote the formation of a microbial community characteristic for BV [2,12].

Recent studies with *P. copri* [13] and *P. bryantii* [14] reveal important catabolic roles of the Na^+^-translocating NADH:quinone oxidoreductase (NQR) and the quinol:fumarate oxidoreductase (QFR) in these *Prevotella* species found in the intestinal tract. Here, we study growth, membrane potential formation, and ammonia production by *P. bivia*. It is demonstrated that the energy metabolism of *P. bivia* relies on NQR and QFR for the recycling of NAD^+^ during growth on glucose. *P. bivia* readily converts asparagine to ammonium, providing endogenous fumarate as an electron sink. The relevance of these findings for the vaginal microenvironment is discussed.

## 2. Results

### 2.1. CO_2_-Dependent Succinate Formation by P. bivia

**Figure 1:**
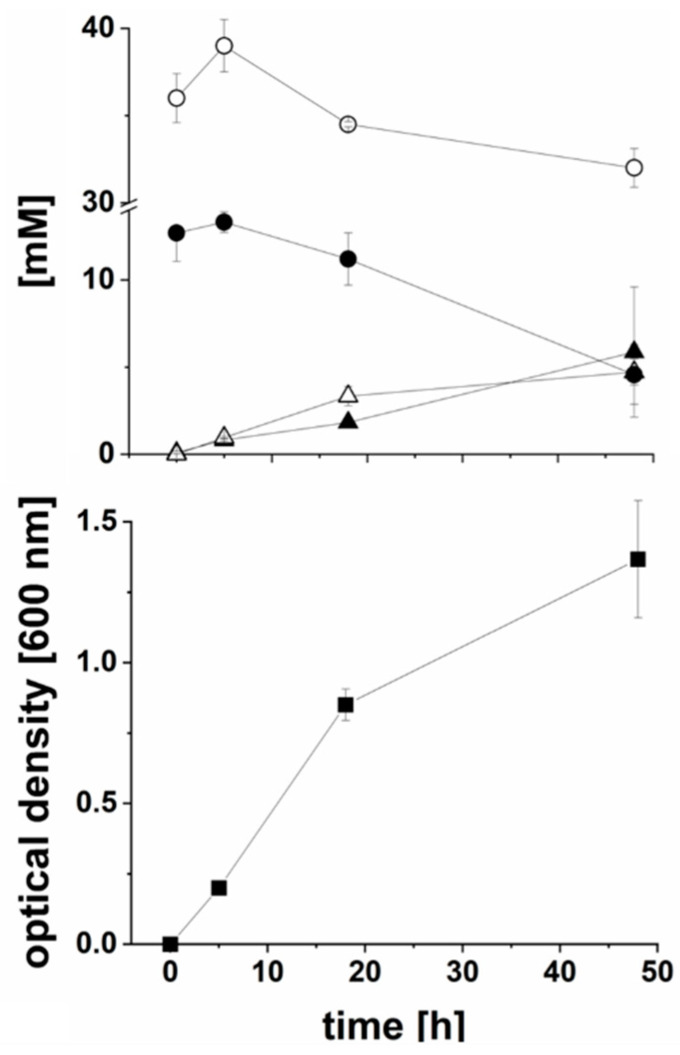
Consumption of glucose and formation of carboxylic acids during anaerobic growth of *P. bivia*. Upper panel: concentrations of glucose (closed circles), acetate (open circles), succinate (closed triangles), and malate (open triangles). Lower panel: optical density at 600 nm. Average and standard deviations of three biological replicates are shown.

*P. bivia* was grown in a synthetic, carbonate-buffered medium (pH 7.5) containing short-chain fatty acids (SCFA’s), glucose, and mixed amino acids (tryptone) [15]. Searching the genome of *P. bivia* DSM 20514 (NCBI accession number: NZ_AJVZ00000000) revealed a putative metabolic route for glucose fermentation involving phosphoenolpyruvate (PEP) carboxykinase and pyruvate oxidoreductase (POR), ultimately leading to succinate and acetate. To test this assumption, *P. bivia* was cultivated and, at indicated times (t = 0, 5, 18, and 48 h), aliquots were retrieved for metabolite analysis by 1D ^1^H NMR. During two days of growth, cell density increased to an OD_600_ of 1.4 ± 0.2 and the glucose concentration decreased from 13 mM to 5 mM (Figure 1), indicating that glucose (8 mM) was utilized as a carbon source by *P. bivia*. Notably, growth during the first 5 h was not accompanied by a decrease in glucose concentration. As previously reported for *P. bivia* grown in vaginal-defined medium [16], succinate (6 mM) was formed as a major product together with malate (5 mM). This indicated a metabolic pathway leading from PEP to oxaloacetate, malate, fumarate, and, finally, succinate.

Short-chain fatty acids including acetate are important growth supplements of *P. bryantii* [15] and were also added to the synthetic growth medium used here. The acetate concentration at the timepoint of inoculation was 36 mM. We observed an increase of acetate by 3 mM during the first 5 h of growth, followed by a decrease to a concentration of 32 mM acetate after 2 d when the cells approached the stationary phase (Figure 1, upper panel). This indicated an initial formation and later an uptake as well as consumption of acetate by *P. bivia*. Formation of acetate starts from PEP, which is converted to pyruvate. Oxidation of pyruvate to acetyl-CoA, conversion to acetyl phosphate, and its reaction with ADP leads to ATP and acetate. Degradation of acetate requires its activation to acetyl-CoA. In *E. coli* [17], this is achieved with the help of the AMP-forming acetyl-CoA synthase. A homolog of this enzyme is found in *P. bivia* (Supplementary Table S1).

**Figure 2:**
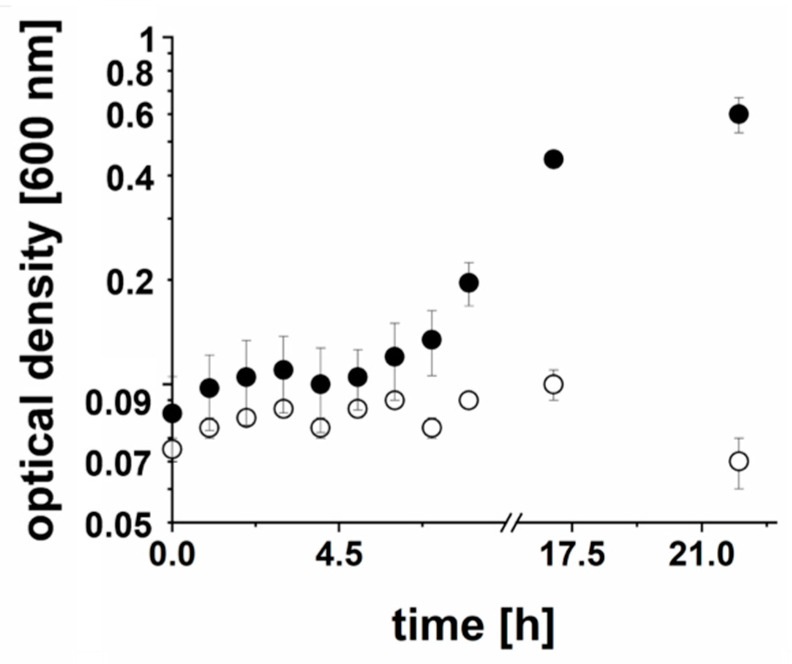
Growth of *P. bivia* is dependent on CO_2_. Cells of *P. bivia* were cultivated in medium prepared with CO_2_ (back circles) or with N_2_ (white circles). Average and standard deviation of three biological replicates are shown.

*P. bivia* did not grow in the medium prepared with N_2_ instead of CO_2_ (Figure 2). This indicates that glucose utilization by *P. bivia* critically depends on carboxylation of PEP by PEP carboxykinase. This CO_2_-dependent step yields oxaloacetate, which is further converted in consecutive steps to malate and fumarate. Fumarate is then reduced to succinate by quinol:fumarate reductase (QFR), as described below. PEP conversion to succinate is a major route in glucose degradation by *P. bivia*. Enzymes catalyzing these reactions, as predicted by genome analysis, are listed with their UNIPROT accession numbers in the electronic Supplementary Material (Table S1).

### 2.2. Ammonia Formation from L-Asparagine by P. bivia

Another important metabolic reaction in *P. bivia* is the conversion of amino acids. A genome search suggested that *P. bivia* might degrade L-asparagine to ammonia with the help of L-asparaginase and aspartate ammonia lyase (Supplementary Material Table S1). L-asparaginase converts L-asparagine into NH_3_ and L-aspartate, and the latter is converted to fumarate and NH_3_ by the aspartate ammonia lyase. High activities of both enzymes were detected in the soluble fraction of *P. bivia*, exhibiting L-asparaginase activity of 951.4 ± 22.3 nmol min^−1^ mg^−1^ and aspartate ammonia lyase activity of 994.9 ± 5.6 nmol min^−1^ mg^−1^.

**Figure 3:**
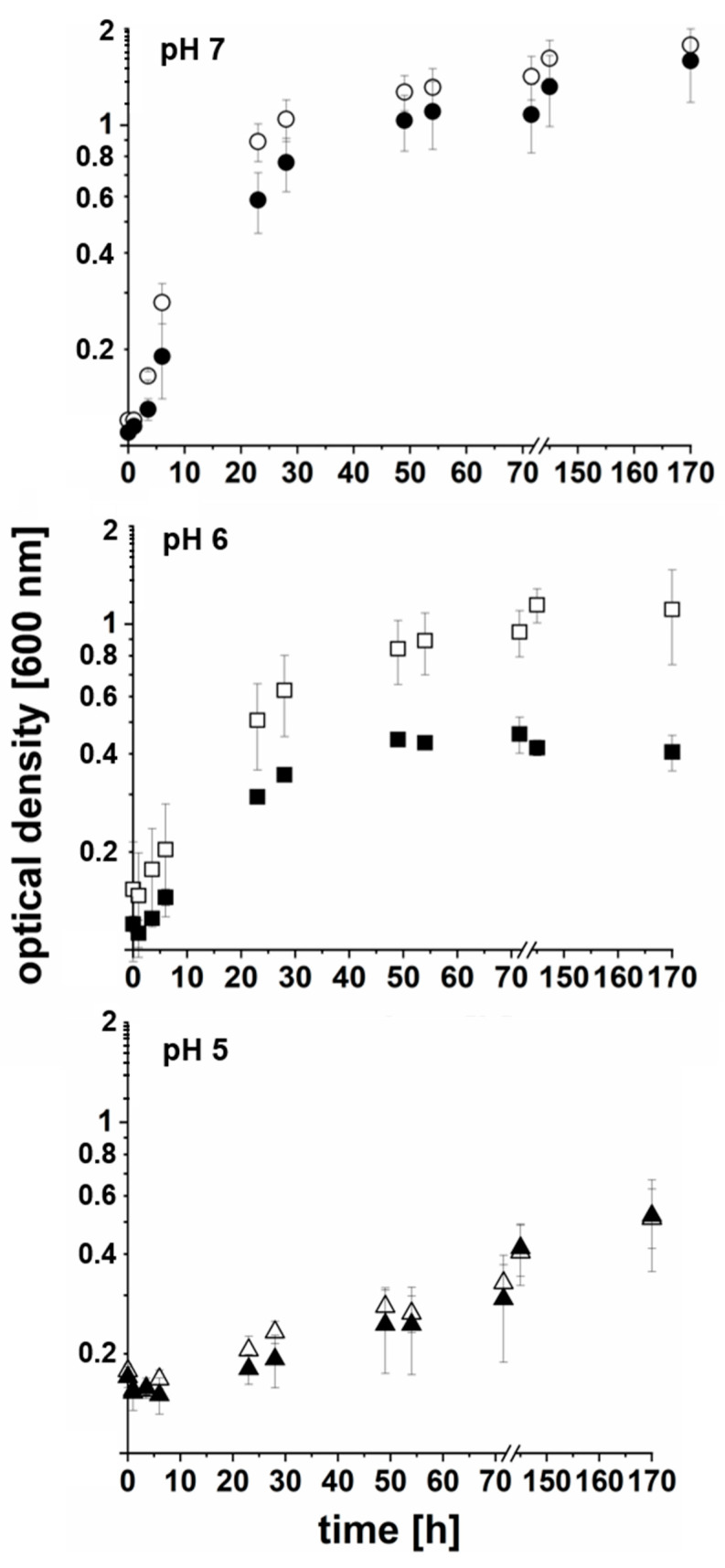
Effect of L-asparagine on the growth of *P. bivia* at varying pH. *P. bivia* was cultivated in medium with (black symbols) or without (white symbols) supplementation of 50 mM of L-asparagine. The initial pH of the medium was 7.0 (circles; top panel), 6.0 (squares; middle panel), and 5.0 (triangles; lower panel). Average and standard deviations of three biological replicates are shown.

The effect and conversion of L-asparagine (50 mM) was also studied with growing *P. bivia* cells in medium that was adjusted to pH 5.0, 6.0, and 7.0 at the timepoint of inoculation. In the controls, asparagine was omitted (Figure 3 and Table 1). The highest growth yield and lowest doubling time was observed at neutral pH without added L- asparagine, with OD_600_ of 1.8 ± 0.2 and 8 h. The addition of Asn had a moderate effect on the final yield (OD_600_ of 1.6 ± 0.4) and doubling time (10 h). This was in marked contrast to the growth at pH 6.0, where the Asn addition led to a decrease in the final yield from OD_600_ = 1.1 ± 0.4 to OD_600_ = 0.4 ± 0.1, and to an increase in doubling time from 13 h to 17 h (Table 1). Very low yield (OD_600_ = 0.5 ± 0.1) and high doubling time (101 h) was observed at pH 5. Here, the Asn addition had no significant effect (Table 1) and yields did not increase further when cells were incubated for two additional days (Figure S1).

**Figure 4:**
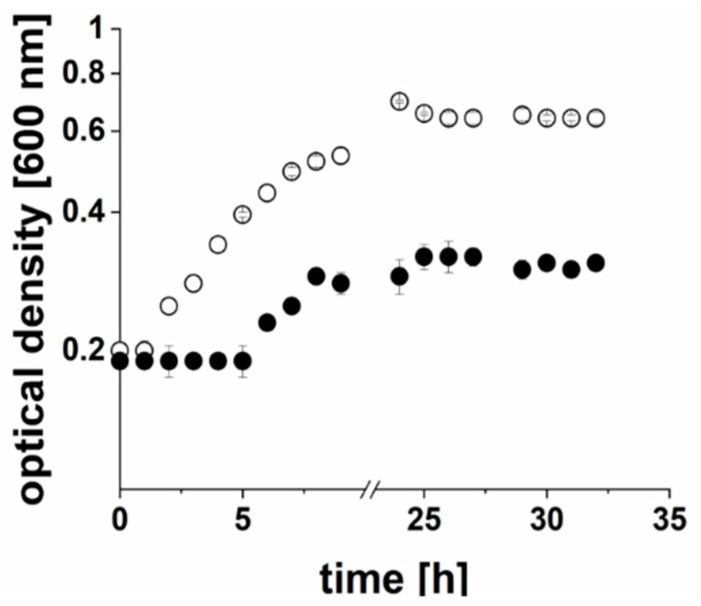
Effect of (NH_4_)_2_SO_4_ on the growth of *P. bivia*. Cells were cultivated with 7 mM of (NH_4_)_2_SO_4_ (white circles) or 80 mM of (NH_4_)_2_SO_4_ (black circles) at pH 6. Average and standard deviations of three biological replicates are shown.

Considering all pH conditions tested, the net formation of ammonium (NH_4_^+^) when 50 mM of asparagine was added to the medium was ~83 mM at pH 7, 75 mM at pH 6, and 48 mM at pH 5 after 7 days (Table 1). Note that at neutral and acidic pH, NH_4_^+^ was the dominant species (>99%). When biomass was taken into account, the highest ammonium formation rate of cells (416.4 ± 31.4 nmol min^−1^ mg^−1^) was observed at pH 6, followed by 265.7 ± 24.1 nmol min^−1^ mg^−1^ at pH 5 and 119.1 ± 2.5 nmol min^−1^ mg^−1^ at pH 7. Without asparagine added, the ammonium concentration in the cultures increased by ~21 mM (pH 7) and ~13 mM (pH 6 and pH 5), suggesting the conversion of amino acids such as asparagine from tryptone, which is a component of the medium. We speculated that the observed reduction of growth at pH 7 and pH 6 in the presence of L-asparagine was caused by the intoxication of cells with NH_3_/NH_4_^+^ [18] formed by *P. bivia*. To test this, *P. bivia* was cultivated at pH 6.0 in the standard growth medium containing 7 mM (NH_4_)_2_SO_4_ or 80 mM (NH_4_)_2_SO_4_. At high (160 mM) NH_4_^+^ concentration, the final cell yields decreased by approximately 50% compared with the cells grown in the presence of 14 mM NH_4_^+^ (Figure 4).

This finding supports the notion that a reduced growth was observed with asparagine at pH 7 and 6, which is caused by the ammonia/ammonium formed from L-asparagine. Notably, asparagine did not influence growth behavior at pH 5.0, although the ammonium formation rate (per mg of cell protein) was higher than at pH 7 (Table 1).

The concentration of succinate in cell-free supernatants from cultures (pH 7.5) in stationary phase (t = 170 h) with and without supplementation of 50 mM of L-asparagine was determined. With 50 mM of L-asparagine added, 38.2 ± 1.5 mM succinate was formed, corresponding to a formation rate of 241.1 ± 9.6 nmol min^−1^ mg^−1^. Without L-asparagine added, 16.1 ± 0.8 mM of succinate was formed, corresponding to a formation rate of 81.1 ± 4.1 nmol min^−1^ mg^−1^. These results indicated that the L-asparagine present in the medium was taken up by *P. bivia* and converted to fumarate, which acted as an electron acceptor by QFR under the formation of succinate.

### 2.3. Membrane-Bound Electron Transfer Complexes in P. bivia

**Figure 5:**
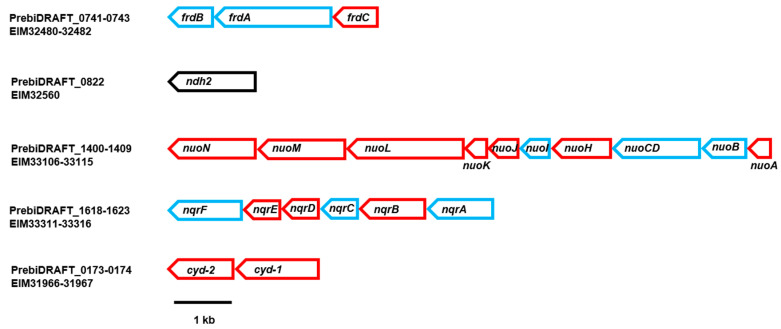
Genes coding for membrane-bound electron transfer complexes of *P. bivia*. ORF (PrebiDRAFT) and NCBI accession numbers (EIM) are given on the left. The fumarate reductase (QFR) was encoded by frdA (1983 bp), frdB (675 bp), and frdC (756 bp). The non-electrogenic NADH dehydrogenase (Ndh2) is a membrane-associated enzyme encoded by ndh2 (1308 bp; black frame). The nuoA (351 bp), nuoB (906 bp), nuoCD (1578 bp), nuoH (1098 bp), nuoI (534 bp), nuoJ (534 bp), nuoK (309 bp), nuoL (2061 bp), nuoM (1512 bp), and nuoN (1440 bp) genes are similar to the genes coding for the corresponding subunits of the 11-subunit complex related to the NUO complex. The Na^+^-translocating NADH:quinone oxidoreductase (NQR) was encoded by nqrA (1359 bp), nqrB (1161 bp), nqrC (714 bp), nqrD (633 bp), nqrE (627 bp), and nqrF (1263 bp). The cytochrome bd quinol oxidase was encoded by cyd-2 (1143 bp) and cyd-1 (1536 bp). Red frames correspond to genes coding for hydrophobic (membrane-bound) subunits. Blue frames correspond to genes coding for hydrophilic (peri or cytoplasmatic) subunits.

**Figure 6:**
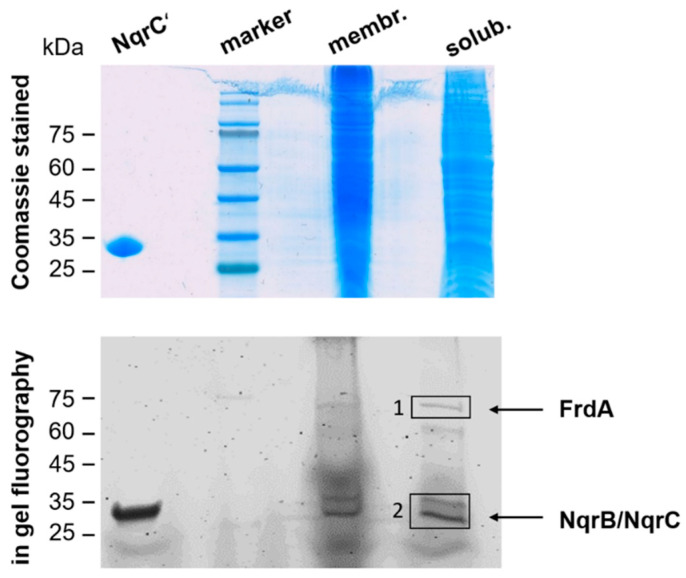
Detection of flavinylated subunits from QFR and NQR in *P. bivia* membranes. Membranes (100 µg; membr.) and membranes solubilized with DDM (100 µg; solub.) of *P. bivia* were separated by SDS PAGE. Proteins were stained with Coomassie (upper panel) and analyzed by in-gel fluorography (lower panel) to detect flavinylated proteins. NqrC’ (25 kDa), the FMN-containing domain of subunit NqrC from *V. cholerae* NQR, served as the control (2 µg). Black boxes (1 and 2) indicate bands subjected to tryptic digestion and mass spectrometry analysis.

The analysis of the *P. bivia* growth medium revealed that succinate is a major product under the chosen conditions, suggesting a reduction of fumarate under the participation of a membrane-bound QFR. The genome of *P. bivia* encodes the FrdABC complex, which is related to the QFR, found in fumarate-respiring anaerobes such as *Wolinella succinogenes* (Figure 5 and Figure S2 in the electronic Supplementary Material). The hydrophilic FrdA subunit is comprised of the fumarate catalytic site and contains one covalently bound FAD [19]. Subunit FrdB, which, similar to FrdA, is oriented towards the cytoplasm, harbors three iron–sulfur centers and interacts with the membrane-bound, quinol-binding FrdC subunit containing two b hemes [19]. Electrons from quinol are transferred from FrdC via FrdB to FrdA, which reduces fumarate to succinate. In-gel fluorography and the subsequent mass spectrometric analysis confirmed the presence of flavinylated FrdA, with an apparent molecular mass of ca. 75 kDa in membranes, and DDM-solubilized membranes of *P. bivia* (Figure 6 and Supplementary Material Tables S2 and S3).

**Figure 7:**
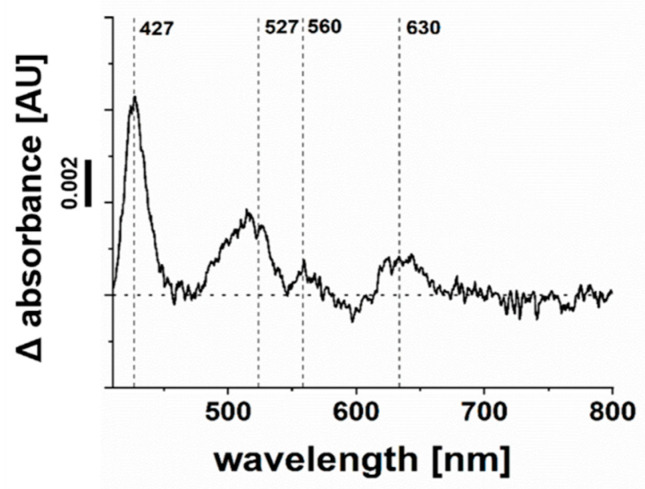
Detection of hemes b and d in solubilized membranes of *P. bivia*. VIS difference spectrum of dithionite-reduced minus air-oxidized DDM solubilisates (0.8 mg protein/mL) with the maxima of reduced heme b (560 nm, 527 nm, and 427 nm) and heme d (630 nm). A typical trace from three biological replicates is presented.

Besides flavins, hemes assigned to QFR and the cytochrome bd quinol oxidase were detected in the VIS redox difference spectrum (dithionite-reduced minus air-oxidized) of solubilized membranes of *P. bivia* (Figure 7). Based on sequence comparison (Figure S2), the FrdC subunit of QFR was predicted to contain two b hemes with absorption maxima at 560 nm, 527 nm, and 427 nm in the reduced state. These typical maxima were detected in the solubilized membranes of *P. bivia*. The maximum at 630 nm (Figure 7) was assigned to heme d of cytochrome bd quinol oxidase [20].

### 2.4. NADH:Quinone and Quinol:Fumarate Oxidoreduction Activities of P. bivia Membranes

As expected from succinate formation and in accord with redox cofactor analyses of membrane proteins, native and DDM-solubilized membranes of *P. bivia* exhibited fumarate reduction activities of 30 ± 2 nmol min^−1^ mg^−1^ and 101 ± 14 nmol min^−1^ mg^−1^, respectively. This raised the question for the redox enzyme providing quinol to QFR. The related *P. bryantii* operates the Na^+^-translocating NADH:quinone oxidoreductase (NQR), feeding redox equivalents from NADH to the quinone pool [14,21]. The NQR is a membrane-bound protein complex composed of six subunits (NqrABCDEF) harboring one FAD, two iron–sulfur centers, one riboflavin, and two covalently bound FMNs [22,23]. The *P. bivia* NQR encoded by the nqr operon (Figure 5) is related to the enzyme from *V. cholerae* (electronic Supplementary Material, Figure S3). In-gel fluorography of *P. bivia* solubilisates revealed two flavinylated proteins running at ~25 kDa (Figure 6). These proteins were assigned to subunits NqrB and NqrC of NQR by mass spectroscopic analysis of the corresponding bands (Tables S2 and S3, Supplementary Material).

*P. bivia* NqrC and NqrB subunits exhibit 50% and 55% sequence identity to the corresponding subunits from *V. cholerae* NQR, including the conserved threonine residues Thr209 (NqrC) and Thr204 (NqrB, *P. bivia* numbering) for covalent attachment of FMNs [24]. *P. bivia* membranes exhibited specific activities of 170 ± 5 nmol min^−1^ mg^−1^ NADH oxidation and 74 ± 6 nmol min^−1^ mg^−1^ ubiquinone-1 (Q1) reduction. DDM-solubilized membranes of *P. bivia* exhibited specific activities of 244 ± 4 nmol min^−1^ mg^−1^ NADH oxidation and 106 ± 20 nmol min^−1^ mg^−1^ Q1 reduction. *P. bivia* harbors genes coding for enzymes that are required for menaquinone synthesis (Men pathway, [25]) but lacks the pathway for ubiquinone synthesis. With 2,3-dimethyl-1,4-naphthoquinone (DMN) as an electron acceptor, *P. bivia* membranes exhibited specific activities of 150 ± 4 nmol min^−1^ mg^−1^ NADH oxidation and 28 ± 10 nmol min^−1^ mg^−1^ DMN reduction. DDM-solubilized membranes of *P. bivia* exhibited specific activities of 287 ± 7 nmol min^−1^ mg^−1^ (NADH oxidation) and 40 ± 1 nmol min^−1^ mg^−1^ (DMN reduction). In *P. bivia*, ORFs assigned to nuo genes suggested the presence of the 11-subunit complex related to the NUO complex (NADH dehydrogenase I, or complex I; Figure 5) [26]. The 11-subunit complex of *P. bivia* lacks the NADH-oxidizing part of the bona fide NUO complex and does not catalyze NADH oxidation. In contrast, the non-electrogenic NADH dehydrogenase (NDH2) [27] encoded by ndh2 (Figure 5) exhibits NADH:Q oxidoreduction activity. To estimate the contribution of NQR and NDH-2 to the overall NADH oxidation activity, the effects of Ag^+^ (an inhibitor of NQR) [14,28] and Na^+^ (the coupling cation of NQR) [23] on NADH:Q oxidoreduction activity were studied. Half-maximal inhibition of NADH oxidation activity was observed at 1 µM Ag^+^, which is reminiscent of the inhibition profile observed with membrane-bound NQR from *Vibrio alginolyticus* [28,29].

**Figure 8:**
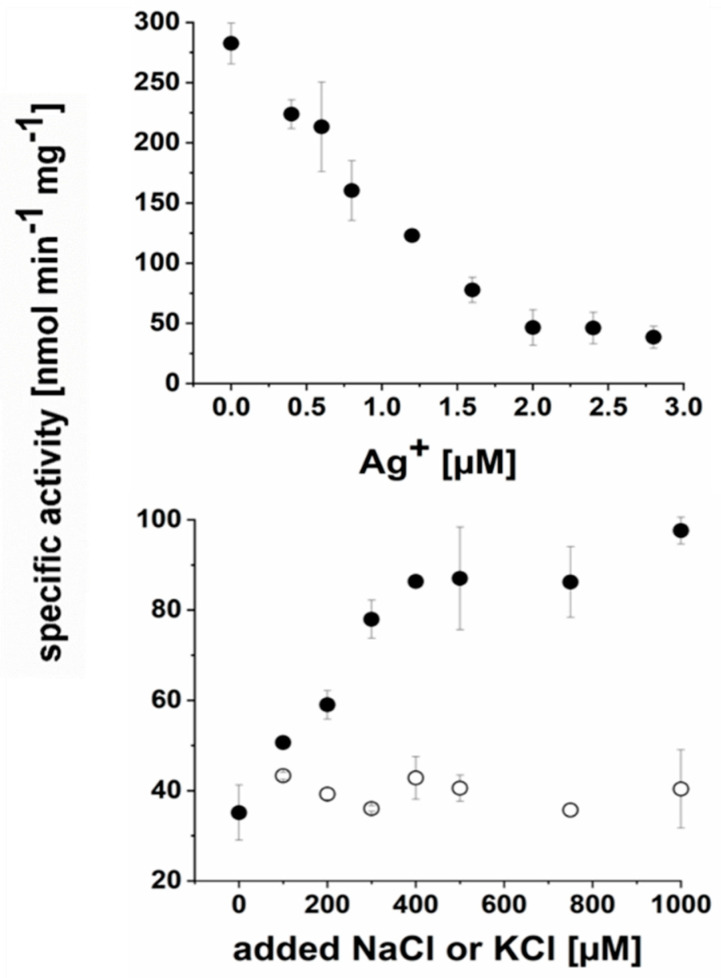
The Na^+^ -translocating NADH:quinone oxidoreductase is the major membrane-bound NADH dehydrogenase in *P. bivia*. Assays were performed with solubilized membranes (50 µg of protein). Upper panel: NADH dehydrogenase activity at increasing [Ag^+^] in chloride-free assay buffer. Lower panel: Q1 reduction activities at increasing [K^+^] (open circles) or [Na^+^] (closed circles). Residual Na^+^ concentration of buffer was at ~10 µM. Average and standard deviations from two technical replicates are shown.

NADH:Q oxidoreduction activity was stimulated by Na^+^, whereas the addition of K^+^ did not lead to increased activity (Figure 8). It is concluded that respiratory NADH oxidation in *P. bivia* is predominantly catalyzed by the Na^+^-translocating NQR. This raised the question regarding whether the formation of a membrane potential in *P. bivia* is stimulated by Na^+^.

### 2.5. Sodium Dependent Membrane Potential in P. bivia

**Figure 9:**
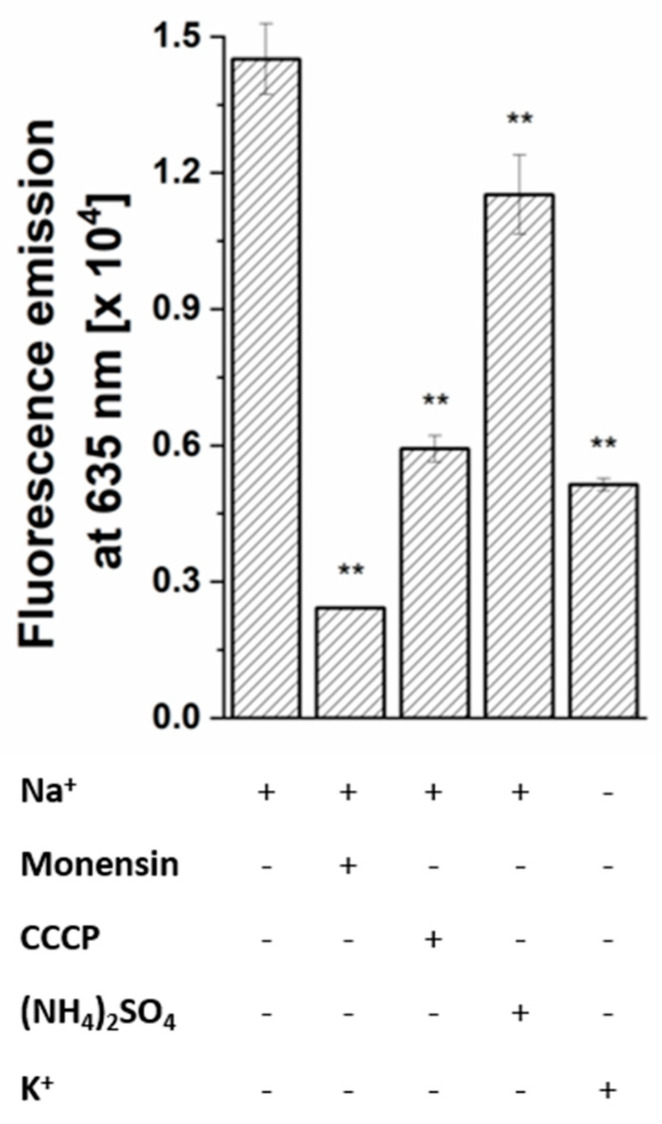
Effect of Na^+^ and uncouplers on the membrane potential of *P. bivia*. The fluorescence emission of cells incubated under the indicated conditions was corrected by the emission of fluorophore in buffer. CCCP, carbonyl cyanide m-chlorophenylhydrazone. Mean values and averages from four technical replicates are shown. Asterisks (**) indicate significant differences from cells incubated with Na^+^ in the absence of inhibitors with *p* < 0.05.

The membrane potential (ΔΨ, inside negative) was estimated using the fluorescent dye DiOC_2_ (3,3′-diethyloxacarbocyanine iodide), which exhibits increased emission at 635 nm in cells with high ΔΨ. The membrane potential established by *P. bivia* was strongly diminished when cells were depleted for Na^+^ by repeated washing with K^+^ (Figure 9). The sodium ionophore monensin, the protonophore carbonyl cyanide m-chlorophenylhydrazone (CCCP), and NH_4_^+^ diminished the membrane potential with decreasing efficiency. This indicates that *P. bivia* maintains an electrochemical Na^+^ gradient (sodium motive force (SMF)). In addition, an electrochemical proton potential (proton motive force (PMF)) was established in *P. bivia*, as indicated by the partial dissipation of the membrane potential by a protonophore, specifically CCCP. In *P. bivia*, ammonium (10 mM) acted as uncoupling agent. This was unexpected given that ammonium in the millimolar concentration range is usually added as a nitrogen source the to bacterial growth media. These findings were in line with the observed reduced growth when Asn or NH_4_^+^ was added to *P. bivia* cultures.

### 2.6. Cytochrome bd Quinol Oxidase of P. bivia

**Figure 10:**
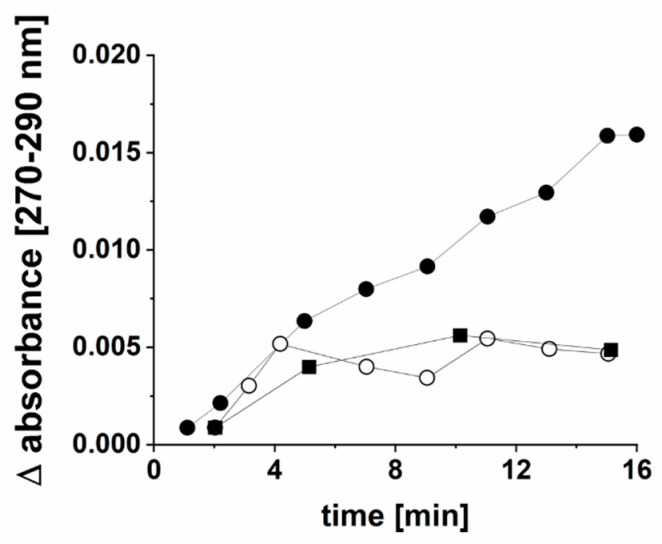
Cytochrome bd quinol oxidase activity of *P. bivia*. Oxidation of DMNH_2_ in the presence of H_2_O_2_ (peroxidase activity) was followed with solubilized membranes from *P. bivia* (closed circles) or *P. bryantii* (open circles). The control reaction was performed in the absence of solubilized membranes (closed squares).

Absorbances in the VIS difference spectrum assigned to heme d and the presence of both cyd-1 and cyd-2 genes suggested the presence of cytochrome bd quinol oxidase in *P. bivia*. This was analyzed by monitoring the peroxidase activity of this enzyme. Solubilized membranes catalyzed the oxidation of the DMNH_2_ with H_2_O_2_ as an electron acceptor with a specific activity of 0.6 nmol min^−1^ mg^−1^. Solubilized membranes from *P. bryantii*, which lack the bd quinol oxidase, exhibited only residual DMNH_2_ oxidation activity at rates similar to protein-free controls (Figure 10).

## 3. Discussion

**Figure 11:**
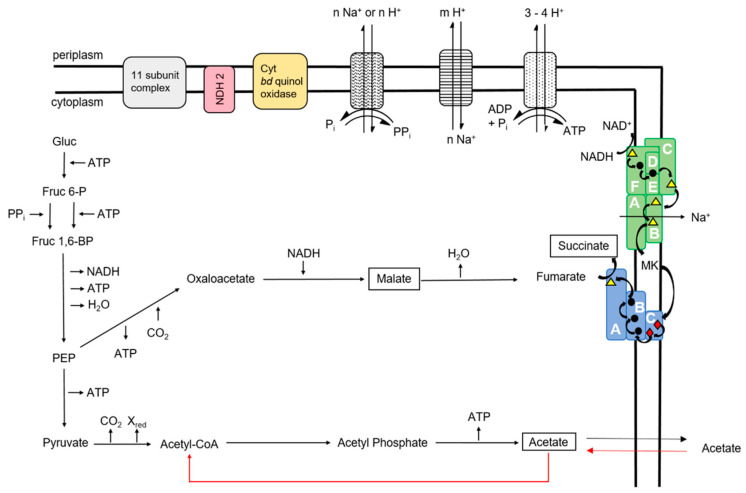
Energy-converting complexes and central carbon metabolism of *P. bivia*. Important products are highlighted by black boxes. Red arrows indicate reactions for the assimilation of acetate. Gluc = glucose; Gluc 6-P = glucose 6-phosphate; and PEP = phosphoenolpyruvate. Blue = fumarate reductase (QFR); green = Na^+^-translocating NADH:quinone oxidoreductase (NQR); dotted = F_1_F_O_ ATPase; striped = Na^+^/H^+^ antiporter; wavy = pyrophosphatase; yellow = cytochrome *bd* quinol oxidase; red = NDH2; and grey = 11-subunit complex related to NDHI (complex I). Subunits of NQR (A–F) and QFR (A–C) are indicated. Colored symbols in the protein complexes represent cofactors. Yellow triangle = flavin; black circle = iron-sulfur center; red diamond = heme *b*; and MK = menaquinone. UNIPROT numbers of proteins are listed in Table S1 (electronic Supplementary Material).

Key metabolic features of *P. bivia* are succinate production, the generation of an electrochemical sodium gradient, the operation of a terminal oxidase, and the conversion of asparagine under the formation of ammonium. From the genome, *P. bivia* is predicted to operate both the typical and atypical Embden-Meyerhoff-Parnas (EMP) pathways, yielding PEP as a central intermediate [30] (Figure S4). The CO_2_-dependent growth indicates that carboxylation of PEP to oxaloacetate by the carboxykinase is crucial for *P. bivia*. This reaction ultimately provides endogenous fumarate, acting as an acceptor for an electron transport chain, which generates a membrane potential (Figure 11). Fumarate is reduced to succinate by the membrane-bound quinol:fumarate oxidoreductase (QFR), which uses menaquinol as a substrate. The product pattern observed with *P. bivia* compares favorably with the results of a transcriptome study of the vaginal microbiota of BV patients, which identified pathways leading to succinate and short-chain fatty acids [31].

Besides QFR, *P. bivia* operates a membrane-bound NADH:quinone oxidoreductase (NQR), which provides menaquinol for the fumarate reduction and regenerates the NAD^+^ required for glycolysis. The similarity of *P. bivia* NQR to the Na^+^-translocating *V. cholerae* NQR [23] and the stimulation of NADH:quinone oxidoreduction activity by Na^+^ indicate that the *P. bivia* NQR acts as a Na^+^ pump. In accordance with this notion, the membrane potential established by *P. bivia* cells critically depends on Na^+^ and collapses in the presence of the sodium ionophore monensin. It is proposed that the build-up of a membrane potential by the Na^+^-translocating NQR is crucial for the energy conservation in *P. bivia*. NQR is widely distributed in *Prevotella* sp. [14] but it is neither found in the *Lactobacilli* of the vaginal microbiota, nor in the human host. This makes NQR an attractive target for the development of antibacterial compounds, as demonstrated for the case of *Chlamydia trachomatis* [32].

Partial dissipation of the transmembrane voltage by the protonophore CCCP indicates that *P. bivia* also establishes a PMF, probably with the help of the F_1_F_O_ ATPase. Considering critical, conserved residues in the cation-binding site [33], the F_1_F_O_ ATPase of *P. bivia* is a proton rather than a sodium-dependent enzyme. To regulate cytoplasmic proton and Na^+^ concentrations, *P. bivia* operates Na^+^/H^+^ antiporters related to NhaA and NhaD (Supplementary Material Table S1; Figure 11).

The formation of H_2_O_2_ and other reactive oxygen species by *Lactobacilli* colonizing the vaginal epithelium [34] prohibits the growth of strict anaerobes, which typically lack enzymes protecting against oxidative stress. *P. bivia* is an exception since it operates a superoxide dismutase [35] and possesses an active bd oxidase utilizing quinol as an electron donor for the reduction of O_2_ or H_2_O_2_. Moreover, *P. bivia* exhibits robust growth at acidic pH, producing ammonium from asparagine. Thus, it is capable of thriving in a vaginal environment dominated by *Lactobacilli.*

BV is characterized by a biofilm [36] established by a microbial consortium, with *Gardnerella vaginalis* and *Prevotella bivia* as prominent strains. In metabolic cross-feeding, ammonium released by *P. bivia* was utilized by *G. vaginalis* [12], followed by the degradation of the vaginal mucus layer by sialidases and adherence of other BV-associated bacteria [37]. *G. vaginalis* lacks metabolic routes for amino acid synthesis with the exception of pathways for the synthesis of L-aspartate and L-asparagine [38]. *P. bivia* possesses L-asparaginase and aspartate ammonia lyase, producing [NH_3_ + NH_4_^+^] at high rates in vitro and in vivo. NH_4_^+^ (160 mM) inhibited the growth of *P. bivia*, most likely due to the partial dissipation of the membrane potential. This is in marked contrast to the situation in *E. coli*, where no detrimental effect on growth was observed up to 500 mM NH_4_^+^ [18]. In a consortium with *G. vaginalis* consuming ammonium, high turnover of Asn by *P. bivia* under the formation of fumarate is possible and *P. bivia* will benefit from fumarate acting as an electron acceptor. This could facilitate the colonization of the vaginal epithelium by *G. vaginalis* and *P. bivia* at an early stage of BV.

## 4. Materials and Methods

### 4.1. Bacterial Strains and Growth Conditions

*Prevotella bivia* DSM 20514 and *Prevotella bryantii* B_1_4 were cultivated anaerobically at 39 °C in a synthetic medium composed of 1% tryptone (w/v), 13 mM of glucose, 50 mM of NaHCO_3_, 15% (by volume) mineral solution 1 (17 mM K_2_HPO_4_), 15% (by volume) mineral solution 2 (17 mM KH_2_PO_4_, 45 mM of (NH_4_)_2_SO_4_, 100 mM of NaCl, 5 mM of MgSO_4_, and 5.4 mM CaCl_2_), and 0.44 μM of resazurin (sodium salt). The redox potential was adjusted with 8 mM of L-Cysteine HCl. In addition, the medium contained (by volume) 0.17% acetic acid, 0.01% n-valeric acid, 0.01% iso-valeric acid, 0.03% n-butyric acid, 0.01% iso-butyric acid, and 0.06% propionic acid [15]. Hungate tubes (7 mL volume) and serum bottles (0.1 L or 1 L volume) with gas-tight caps were used.

### 4.2. Isolation and Solubilization of Membranes

Cells were harvested at an OD_600_ of 1.5–2.0 (*P. bivia*) or at an OD_600_ of 2.5–3.0 (*P. bryantii*) by centrifugation at 9000× *g* for 30 min (4 °C). The cells were washed twice in 20 mM of Tris-H_2_SO_4_ (pH 7.5) and 50 mM of K_2_SO_4_. Cells (10 g wet weight) were resuspended in 30 mL of 20 mM Tris-H_2_SO_4_ (pH 7.5) containing 50 mM of K_2_SO_4_, 5 mM of MgSO_4_, 1 mM of dithiothreitol, 1 mM of phenylmethyl sulfonylfluoride (PMSF), 0.1 mM of diisopropyl fluorophosphate, and traces of DNase I (Roche Diagnostics GmbH, Mannheim, Germany). The suspension was passed three times through an EmulsiFlex^®^-C3 high-pressure homogenizer (Avestin Europe GmbH, Mannheim, Germany) at 20,000 psi. Cell debris and unbroken cells were removed by centrifugation at 27,000 g for 30 min at 4 °C. Membranes were collected by ultracentrifugation (50,000 rpm, Beckman Ti70 rotor; Beckman Coulter GmbH, Krefeld, Germany) for 90 min at 4 °C; washed once in 20 mM of Tris-H_2_SO_4_ (pH 7.5), 50 mM of K_2_SO_4_, and 5% (*v*/*v*) glycerol; and resuspended in the same buffer. The membrane suspension (5–10 mg protein/mL) was frozen by pipetting aliquots of 30 μL into liquid N_2_. The frozen droplets were collected and stored in liquid N_2_ until further use. For the solubilization of the membranes, protein and n-dodecyl-ß-D-maltoside (DDM; 7.5 µM final concentration) were incubated in a 1:3.75 (protein:detergent) ratio in a buffer containing 20 mM of Tris-H_2_SO_4_ (pH 7.5), 50 mM of K_2_SO_4_, and 5% (*v*/*v*) glycerol, with a total volume of 1.5 mL, for 2 h at 6 °C under gentle shaking (350 rpm). The membrane suspensions were ultracentrifuged (50,000 rpm, Beckman Ti70 rotor; Beckman Coulter GmbH, Krefeld, Germany) for 45 min at 4 °C. Supernatants containing solubilized membrane proteins were frozen and stored in liquid N_2_ as described above.

### 4.3. Bacterial Growth

Growth was followed in Hungate tubes with 7 mL of medium inoculated with 500 µL of *P. bivia* overnight culture grown at pH ~7. Turbidity of the cultures in tubes was measured with a cell density meter (WPA biowave CO8000, Biochrom Ltd., Cambridge, UK) at 600 nm. To analyze the CO_2_ dependency of the growth of *P. bivia*, triplicate growth experiments with medium prepared with CO_2_ [15] were conducted. In the controls, CO_2_ was replaced with N_2_. To analyze the medium during growth under a chosen condition, six tubes per experiment were inoculated at t = 0 h and turbidity was monitored in parallel. At indicated times, the culture from one tube was retrieved. To study the effect of pH on the growth of *P. bivia*, the growth medium was adjusted to pH 5, 6, or 7 by adding NaOH. Growth was monitored for 7 days (pH 6 and pH 7) or 9 days (pH 5) in triplicate experiments. At indicated times, cells were harvested by centrifugation at 16,000 g for 5 min at 4 °C and both pH and ammonium concentration of supernatants were determined. In parallel growth experiments, the media contained 50 mM of L-asparagine. To study the effect of NH_4_^+^ on growth, medium (pH 6.0) was supplemented with 7 mM of (NH_4_)_2_SO_4_ and 14 mM of K_2_SO_4_, or with 80 mM of (NH_4_)_2_SO_4_ and 20 mM of K_2_SO_4_. To identify and quantify organic compounds in cultures by ^1^H-NMR, experiments in Hungate tubes were performed in triplicates. After 5 h, 18 h, and 48 h of growth, the ODs were determined and one culture was harvested by centrifugation at 16,000 g for 5 min at 4 °C to obtain supernatants for NMR analysis.

### 4.4. Analytical Methods

The protein concentration was determined with the bicinchoninic acid method [39] using the reagent from Pierce^TM^ (ThermoFisher Scientific, Waltham, MA, USA). To determine the protein content of the cell suspensions, cells from 1 mL of culture were washed in 300 mM of sucrose. The cell pellet was resuspended in 5% (*v*/*v*) trichloroacetic acid and heated (100 °C) for 10 min [40]. [NH_3_ + NH_4_^+^] in supernatants of cell cultures was determined spectrophotometrically with the Nessler’s reagent [41]. Ammonium sulfate was used as the standard.

Glucose, acetate, succinate, and malate in cultures of *P. bivia* were determined by 1D ^1^H NMR spectroscopy. Supernatants from cell cultures were dried with a vacuum concentrator (program V-AQ; Eppendorf SE, Hamburg, Germany,). The pellets were resuspended in 50 mM of Na_2_HPO_4_ (pH 7) in D_2_O containing 5 mM of 3-trimethylsilyl propionic-2,2,3,3 acid sodium salt (TSP) as an internal reference for the ^1^H chemical shift calibration and the suspensions were filled into NMR tubes. 1D ^1^H NMR spectra were recorded using a Bruker Avance III HD NMR 600 MHz spectrometer equipped with a 5 mm BBO Prodigy cryo-probe (Bruker BioSpin GmbH, Ettlingen, Germany). For structural identification of the metabolites, 1D ^1^H heteronuclear NMR experiments (*g*HSQC and *g*HMBC) [42] were recorded at 298 K. For acquisition, processing, and evaluation of NMR spectra, the software TopSpin 3.5pl7 (Bruker BioSpin GmbH, Ettlingen, Germany) was used. To quantify succinate in medium, to which 50 mM of L-asparagine was added at the start of the growth, cells were cultivated for 50 h in Hungate tubes. Cell-free supernatants were analyzed using the Sigma -Aldrich^TM^ Succinate Assay Kit (Merck KGaA, Darmstadt, Germany).

Denaturing polyacrylamide gel electrophoresis (SDS-PAGE) was performed with a 12% polyacrylamide gel [43]. Protein and membrane suspensions were diluted in 5x SDS sample buffer (500 mM of DTT; 1 M of Tris-HCl, pH 6.8; 5% SDS; and 28.8% glycerol (*w*/*v*), bromophenol blue) and boiled for 5 min before loading on the gel. In-gel fluorescence of covalently bound flavins in NqrB, NqrC, and FrdA, separated by SDS-PAGE, was detected using the ImageQuant LAS 4000 imager (λ_excitation_ = 460, emission filter = Y515 CyTM2; Cytiva, Marlborough, MA, USA). As a positive control, the purified NqrC’ subunit was used. This protein is a truncated variant of the NqrC subunit of the *V. cholerae* NQR comprising the covalently attached FMN but lacking the N-terminal transmembrane helix [44]. The molecular mass of NqrC’ was 25.38 kDa. Proteolysis of proteins separated by SDS-PAGE, followed by mass spectrometric analysis of the peptides, was performed as described previously [21].

### 4.5. UV/Vis Difference Spectra of Redox Cofactors in P. bivia

The absorption spectrum of dithionite-reduced redox cofactors in solubilized membranes of *P. bivia* was compared with an aliquot of the same sample with cofactors in their air-oxidized state using a double-beam UV/VIS spectrophotometer (UV-2600i; Shimadzu GmbH, Berlin, Deutschland). Light is split by a half mirror passing separately through the reference sample (beam 1) and through the test sample (beam 2). The light intensities passing through the sample and reference were compared. The range of 220–800 nm was monitored with an interval of 0.5 nm and with medium scan speed. The difference in absorbance of beam 2 minus beam 1 at a given wavelength was calculated using the software LabSolutions UV-Vis (Shimadzu GmbH, Berlin, Deutschland). DDM-solubilized membranes of *P. bivia* were analyzed at a concentration of ~0.8 mg of protein per mL in 20 mM of potassium phosphate buffer, pH 7.5. Beam 1 contained air-oxidized solubilisate, whereas in beam 2, an aliquot of solubilisate mixed with a few crystals of sodium dithionite was analyzed. The difference spectrum of dithionite-reduced minus air-oxidized membranes was recorded.

### 4.6. Enzymatic Assays

NADH oxidation and quinone reduction activities were monitored simultaneously in a quartz cuvette (d = 1 cm) in a total volume of 1 mL at 25 °C using a Hewlett Packard 8452A diode-array spectrophotometer (Agilent Technologies, Santa Clara, CA, USA). NADH oxidation was followed at 340 nm (ε_NADH_ = 6.22 mM^−1^ cm^−1^) and ubiquinone-1 (Q1) or 2,3-dimethyl-1,4-naphthoquinone (DMN) reduction at 280 nm (ε_Q1_ = 14.5 mM^−1^ cm^−1^ and ε_DMN_ = 15.2 mM^−1^ cm^−1^) [45]. Solubilized membranes of *P. bivia* (50 µg of protein in 20 mM of Tris-H_2_SO_4_, pH 7.5; 50 mM of K_2_SO_4_; 5% (*v*/*v*) glycerol; and 7.5 µM of DDM) were incubated with varying amounts of AgNO_3_ (0–3.0 µM) for 5 min at 4 °C and were added to a cuvette with buffer (20 mM of Tris H_2_SO_4_, pH 7.5; 100 mM of Na_2_SO_4_; 100 µM of NADH; and 100 µM of Q1) to start the enzymatic reaction. In buffers, chloride was replaced with sulfate to avoid precipitation of AgCl. To study the effect of Na^+^ on NADH dehydrogenase activity of solubilized membranes (50 µg), NaCl (0–1000 µM) or corresponding amounts of KCl (0–1000 µM) were added to the assay buffer (20 mM of Tris H_2_SO_4_, pH 7.5; 100 µM of NADH; and 100 µM of Q1). The residual Na^+^ concentration in the assay without the added NaCl was ~10 µM, as determined by atomic absorption spectroscopy (AA240, Agilent Technologies, Santa Clara, CA, USA).

Quinol:fumarate oxidoreductase (QFR) activity was determined with anoxic materials and buffer (20 mM of potassium phosphate, pH 7.5) containing benzyl viologen (~0.5 mM) in the anaerobic chamber. Benzyl viologen was reduced by adding sodium dithionite crystals to achieve an absorbance of 1 at 564 nm [46]. Then, 100–200 μg of protein was added. The reaction was started by adding 10 mM of fumarate. Decrease in absorbance of benzyl viologen was monitored at 564 nm (ε = 19.5 mM^−1^ cm^−1^) in a cuvette at 20 °C using a diode array spectrophotometer (Black-comet, StellarNet Inc., Tampa, FL, USA). The cuvette holder was placed inside the anaerobic chamber. The detector and light source (SL5 UV + VIS lamp, StellarNet Inc., Tampa, FL, USA) were placed outside of the anaerobic chamber and the components were connected with fiber optic cables.

The cytochrome quinol *bd* oxidase activity in solubilized membranes was determined by following the oxidation of quinol with H_2_O_2_ [47]. 2,3-dimethyl-1,4-naphthoquinol (DMNH_2_), obtained as described in [48], was used as an electron donor. DMNH_2_ oxidation with H_2_O_2_ as an electron acceptor was monitored from the formation of DMN (ε_DMN_ = 15.2 mM^−1^ cm^−1^) [49] under anoxic conditions by recording difference spectra over 16 min in a double-beam photometer (Lambda 16, PerkinElmer, Waltham, MA, USA). Buffers and reagents were made anoxic by flushing with N_2_ and were mixed in cuvettes inside the anaerobic chamber. Cuvettes were sealed gas-tight and difference spectra were recorded immediately outside of the chamber. In beam 1, the reference cuvette was analyzed, containing 1 mL of assay solution (180 µg of solubilized membrane protein from *P. bivia* or *P. bryantii*; 50 mM of MOPS, pH 7.0; 100 mM of NaCl; 0.1% *v*/*v* DDM; and 200 µM of DMNH_2_). A cuvette with 1 mL of assay solution mixed with 10 µL of 30% (by volume) H_2_O_2_ was placed in beam 2. To calculate the rates of DMNH_2_ oxidation, the difference in absorbance from 270 nm to 290 nm obtained by subtraction of the spectrum of beam 1 from the spectrum of beam 2 at a given timepoint was determined. In the control, H_2_O_2_ was omitted.

For L-asparaginase and aspartate lyase activity determinations, the soluble protein fraction of *P. bivia* was obtained by ultracentrifugation of crude cell extracts. L-asparaginase activity was determined as described in [41]. Aspartate ammonia lyase activity was determined as described in [50] and modified as follows. After incubation at 30 °C for 30 min, the assay solution (1 mL) was heated at 80 °C for 5 min to stop the reaction. The (NH_3_ + NH_4_^+^) concentration was determined photometrically with the Nessler’s reagent [51].

### 4.7. Membrane Potential

Membrane potential of *P. bivia* was estimated with the BacLight^TM^ Bacterial Membrane Potential Kit ((ThermoFisher Scientific, Waltham, MA, USA)) using a Infinite F200 Pro plate reader (Tecan Deutschland GmbH, Crailsheim, Germany) [52]. *P. bivia* cells were cultivated in Hungate tubes until an OD_600_ of 0.6 was reached. The following steps were performed in the anaerobic chamber. Cells were harvested, diluted in sodium buffer (10 mM of sodium buffer, pH 7.4, and 145 mM of NaCl) or potassium buffer (10 mM of potassium buffer, pH 7.4, and 145 mM of KCl), and adjusted to OD_600_ = 0.25. Cells in 800 µL of this suspension were sedimented by centrifugation (16,000 g, 5 min), washed twice, and resuspended in 800 µL of the corresponding buffer. To analyze the effect of ionophores on the membrane potential, 2.5 µM of carbonylcyanid-m-chlorphenylhydrazon (CCCP), 2.5 µM of monensin, or 5 mM of (NH_4_)_2_SO_4_ were added to cell suspensions, as indicated. After incubation for 10 min (20 °C), the fluorescence dye 3,3′-diethyloxacarbocyanine iodide (DiOC_2_, 15 µM) was added and cells were further incubated for 60 min in the dark. Outside of the anaerobic chamber, three aliquots (200 µM) of each sample were applied to a black, flat-bottom 96-well plate (polysterene; 4titude Ltd., Berlin, Germany). To determine red fluorescence intensities, excitation was set to 480 nm (band width, 9 nm) and emission to 635 nm (band width, 20 nm; gain, 117). To determine green fluorescence intensities, the emission was changed to 535 nm (band width, 25 nm; gain, 107). Fluorescence emission intensities were in the linear range of the fluorescence detector. Background fluorescence intensities of buffer with dye and of cell suspensions were determined for background corrections. As expected, an increase of red fluorescence intensity, indicating a transmembrane voltage, was accompanied by a decrease in green fluorescence intensity. Mean values of red fluorescence intensities are presented.

## Figures and Tables

**Table 1:** Effect of pH and L-asparagine on the growth and formation of (NH_3_ + NH_4_^+^) by *P. bivia*. OD_600_ and [NH_3_ + NH_4_^+^] were determined in the stationary phase (t = 7 d) of *P. bivia* cultures grown at pH 5, 6, or 7 with or without supplementation of 50 mM of L-asparagine.

Growth Condition	OD_600_ (t = 7 d)	Doubling Time (h)	NH_4_^+^ (mM; t = 0)	NH_4_^+^ (mM; t = 7)	Net (NH_3_ + NH_4_^+^) Formed (mM)	Rate of Net (NH_3_ + NH_4_^+^) Formation (nmol min^−1^ mg^−1^)
pH 5 + Asn	0.5 ± 0.1	91	14.7 ± 0.2	62.9 ± 0.8	48.2 ± 4.4	265.7 ± 24.1
pH 5 − Asn	0.5 ± 0.1	101	14.8 ± 0.1	27.9 ± 0.2	13.1 ± 0.1	59.7 ± 0.4
pH 6 + Asn	0.4 ± 0.1	17	16.9 ± 2.1	92.4 ± 1.1	75.5 ± 5.6	416.4 ± 31.4
pH 6 − Asn	1.1 ± 0.4	13	12.1 ± 0.5	25.5 ± 0.2	13.1 ± 0.6	21.7 ± 2.1
pH 7 + Asn	1.6 ± 0.4	10	18.1 ± 0.8	101.5 ± 0.5	83.4 ± 4.1	119.1 ± 2.5
pH 7 − Asn	1.8 ± 0.2	8	16.5 ± 0.7	37.6 ± 0.03	21.1 ± 0.25	25.2 ± 0.5

## Data Availability

Additional data are provided in the Supplementary Materials.

## Supplementary Materials

Supplementary Materials are available online at https://www.mdpi.com/article/10.3390/ijms222111925/s1.
