# Supplementary material for: Central Carbon Metabolism, Sodium-Motive Electron Transfer, and Ammonium Formation by the Vaginal Pathogen Prevotella bivia
Source: Int J Mol Sci. 2021 Nov 3;22(21):11925. doi: 10.3390/ijms222111925 (PMC8585091; doi:10.3390/ijms222111925)
Supplement: Supplementary file 1 [file ijms-22-11925-s001.zip › ijms-1440859-supplementary/Supplementary/supplementary material.pdf]

International Journal of *Molecular Sciences*

MDPI

Electronic supplementary material

Central carbon metabolism, sodium-motive electron transfer and ammonium formation by the vaginal pathogen *Prevotella bivia*

Lena Schleicher[1,2], Sebastian Herdan[1,2], Günter Fritz†[1,2], Andrej Trautmann[2,3], Jana Seifert[2,3], Julia Steuber†[1,2,*]

[1] Institute of Biology, University of Hohenheim, Garbenstraße 30, 70599 Stuttgart, Germany
[2] HoLMiR- Hohenheim Center for Livestock Microbiome Research, University of Hohenheim, Leonore-Blosser-Reisen-Weg 3, 70599 Stuttgart, Germany
[3] Institute of Animal Science, University of Hohenheim, Emil-Wolff-Straße 8, 70599 Stuttgart, Germany
* Correspondence: Julia Steuber, julia.steuber@uni-hohenheim.de, +49 711 459 22228

This PDF files includes:
Figure S1, S2, S3, S4
Table S1, S2

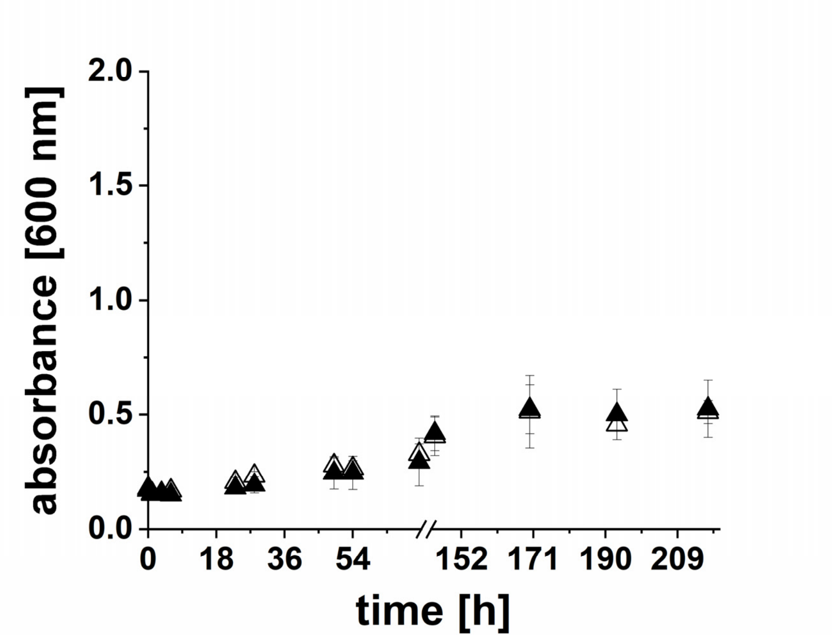

Figure S1. Growth of *P. bivia* at pH 5.0 with (black triangles) or without (white triangles) 50 mM L-asparagine added. Average and standard deviations of three biological replicates are shown.

FrdA

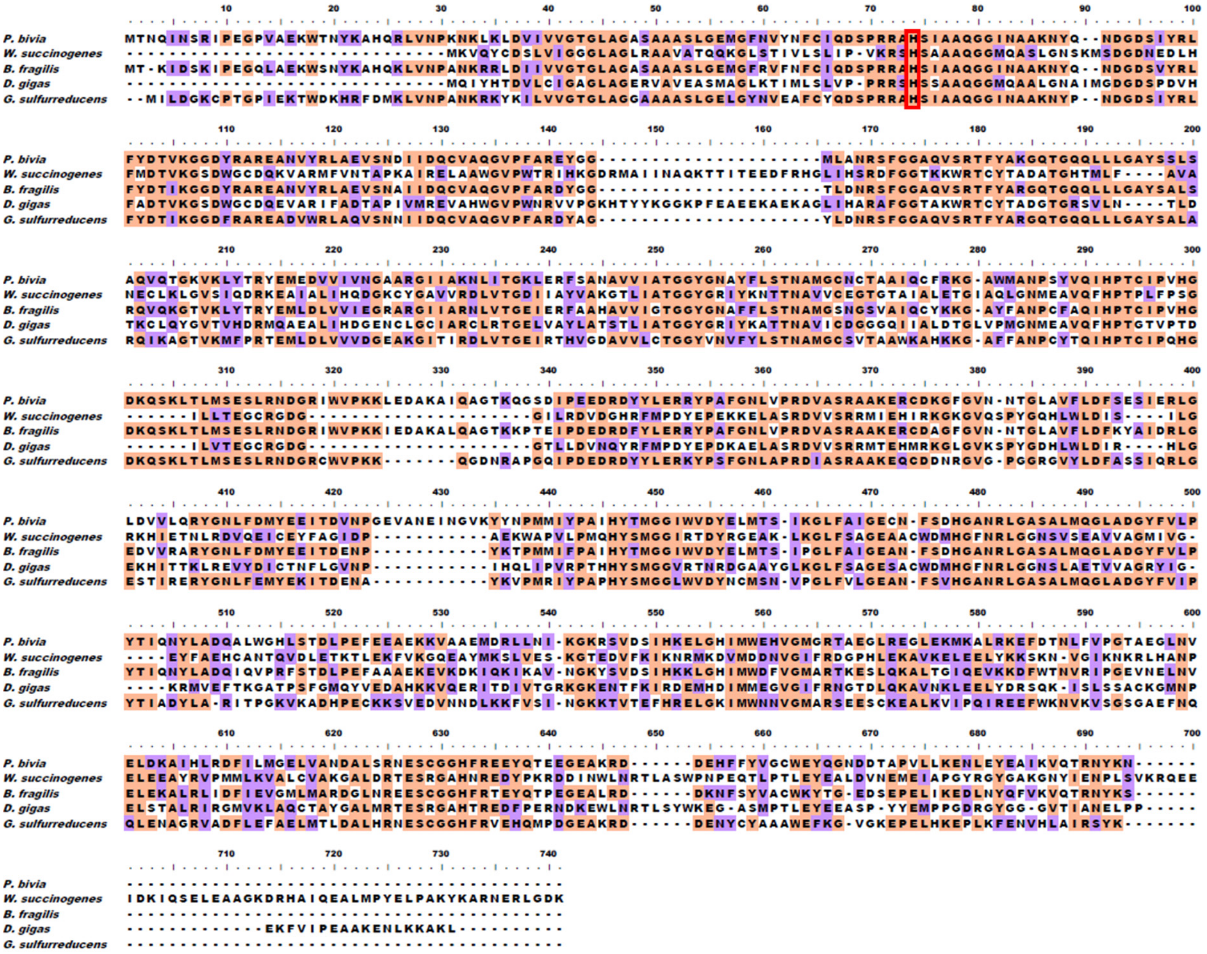

FrdB

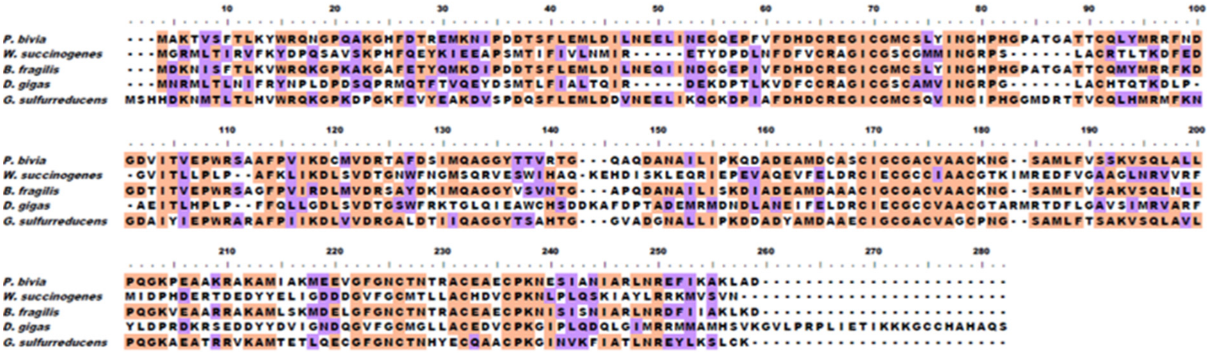

FrdC

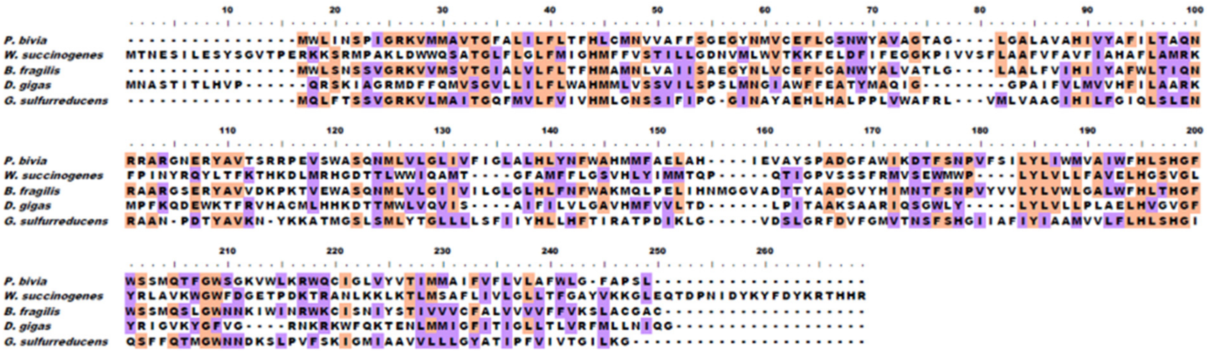

Figure S2. Protein sequence alignments of the QFR subunits FrdA, FrdB and FrdC form *Prevotella bivia, Wolinella succinogenes, Bacteroides fragilis, Desulfovibrio gigas* and *Geobacter sulfurreducens*. Orange, identical amino acids; purple, similar amino acids. Conserved amino acids for flavin binding are highlighted in red.

NqrA

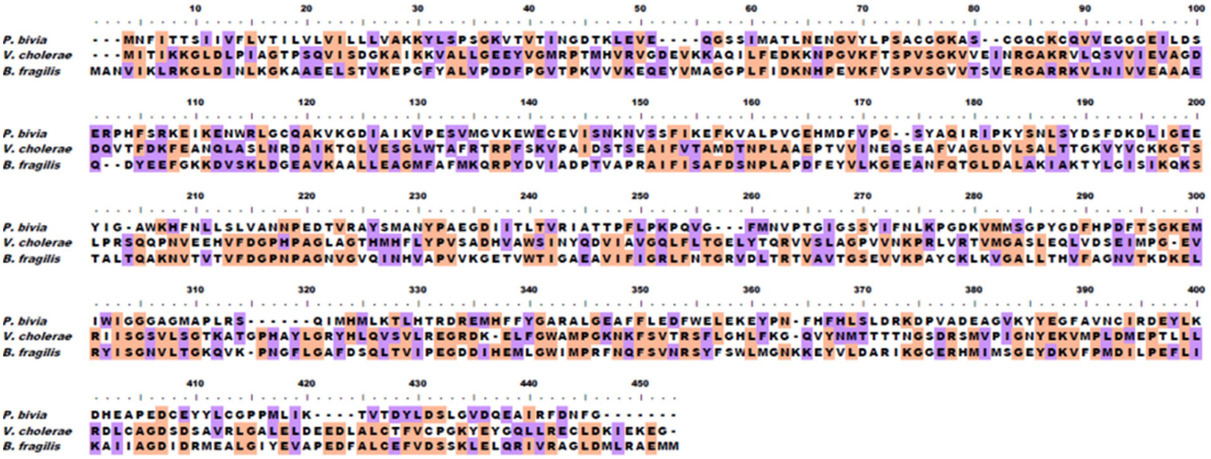

NqrB

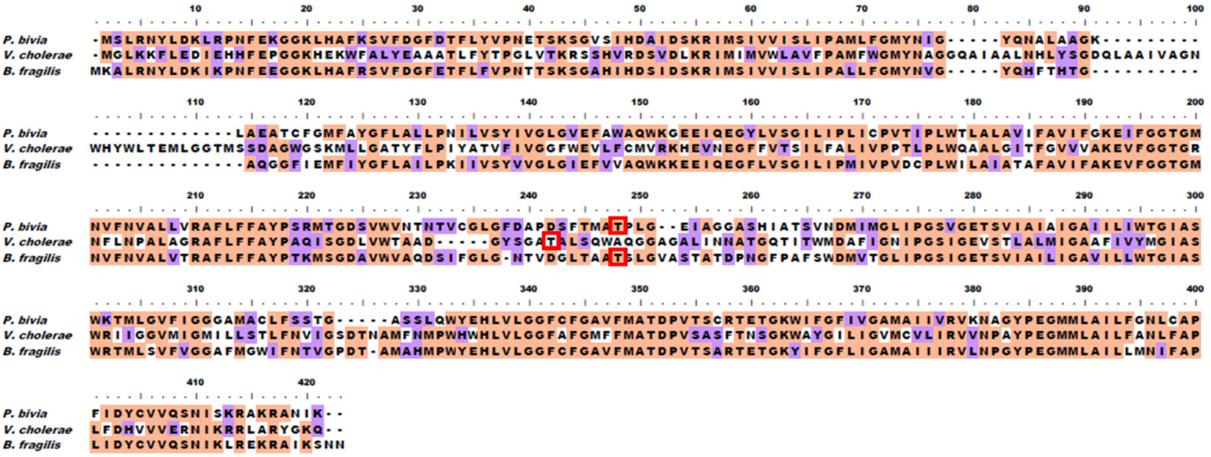

NqrC

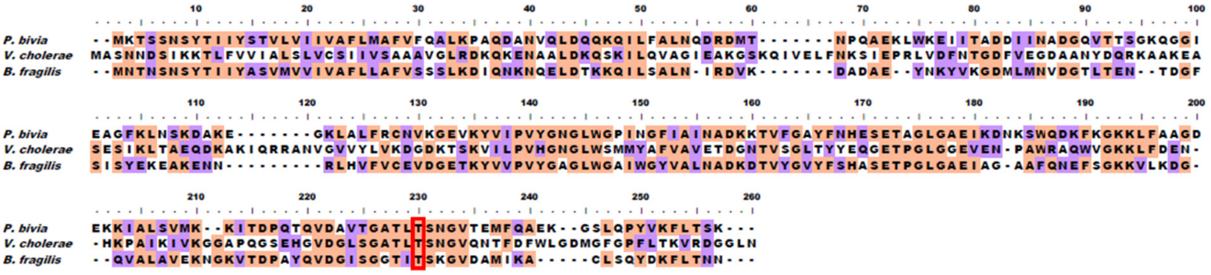

NqrD

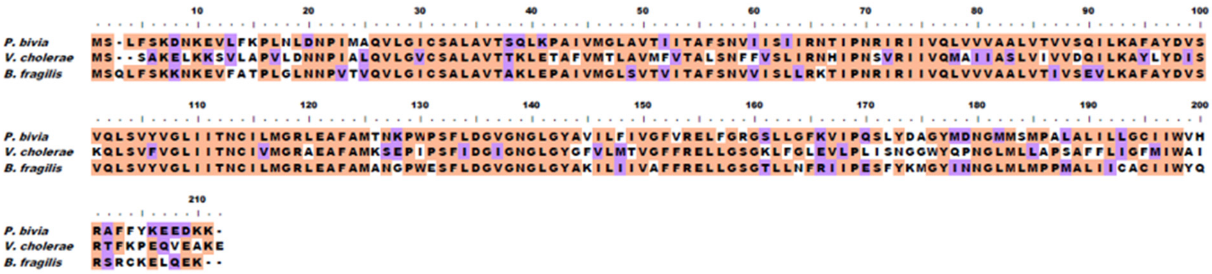

NqrE

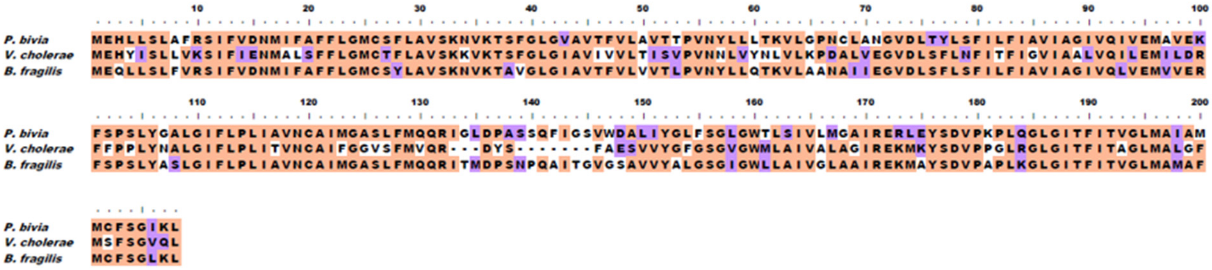

NqrF

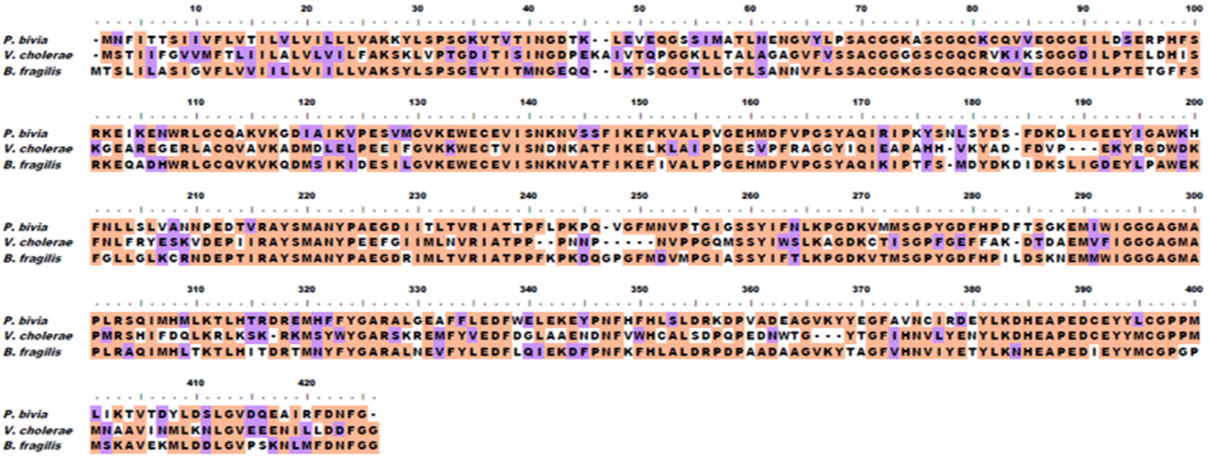

Figure S3. Protein sequence alignments of the NQR subunits NqrA, NqrB, NqrC, NqrD, NqrE and NqrF form *Prevotella bivia, Vibrio cholerae* and *Bacteroides fragilis*. Orange, identical amino acids; purple, similar amino acids. Conserved amino acids for flavin binding are highlighted in red.

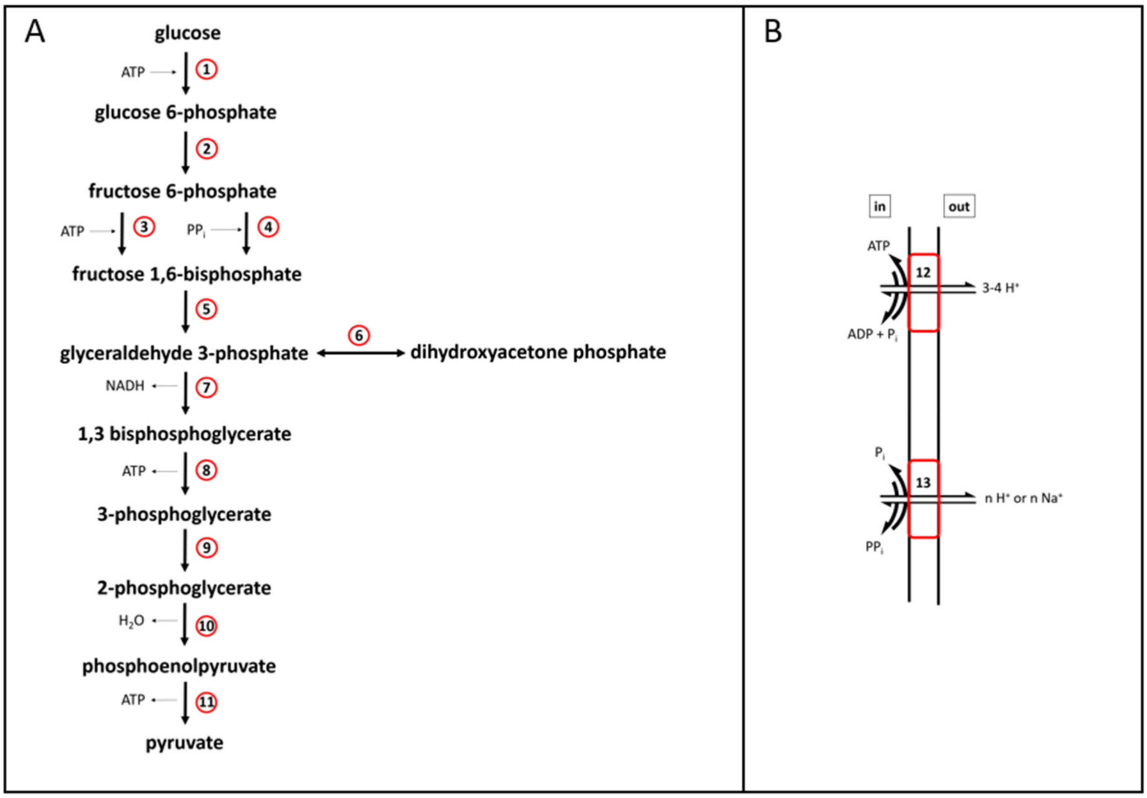

Figure S4. Predicted pathways for glycolysis, and enzymes for regeneration of ATP or PP_i in *Prevotella bivia*. A: Conversion of glucose to pyruvate. Glucokinase (1); glucose 6-phosphate isomerase (2); ATP-dependent phosphofructokinase (3); PP_i-dependent phosphofructokinase (4); aldolase (5); triose-phosphate isomerase (6); glyceraldehyde dehydrogenase (7); phosphoglycerate kinase (8); phosphoglycerate mutase (9); enolase (10); pyruvate kinase (11). B: Regeneration of ATP or PP_i by the F_1F_O ATPase (12) or the pyrophosphatase (13) is driven by electrochemical H^+- or Na^+ -gradients.

Table S1. Enzymes involved in glycolysis and energy conversion of *P. bivia* DSM 20514. Functional assignments are based on the genome information (NCBI accession number; NZ_AJVZ00000000). Corresponding UNIPROT accession numbers and predicted catalytic activities are listed.

Enzyme	UNIPROT acccession number	Reaction
L-asparaginase	I4ZB31	L-asparagine + H_2O → L-aspartic acid + NH_3
Aspartate ammonia lyase	I4ZB31	L-aspartic acid ⇌ fumarate + NH_3
Glucokinase	I4Z6R6	glucose + ATP → glucose 6-phosphate + ADP + P_i
Glucose 6-phosphate isomerase	I4Z9B5	glucose 6-phosphate ⇌ fructose 6-phosphate
ATP-dependent phosphofructokinase	I4ZBC7	fructose 6-phosphate + ATP → fructose 1,6-bisphosphate + ADP + P_i
PP_i-dependent phosphofructokinase	I4Z6W8	fructose 6-phosphate + PP_i → fructose 1,6-bisphosphate + P_i
Aldolase	I4Z941	fructose 1,6-bisphosphate → glyceraldehyde 3-phosphate + dihydroxyacetone phosphate
Triose-phosphate isomerase	I4ZC94	dihydroxyacetone phosphate ⇌ glyceraldehyde 3-phosphate
Glyceraldehyde dehydrogenase	I4ZAB6	glyceraldehyde 3-phosphate + NAD^+ + P_i ⇌ 1,3-bisphosphoglycerate + NADH + H^+
Phosphoglycerate kinase	I4ZC16	1,3-bisphosphoglycerate + ADP + P_i ⇌ 3-phosphoglycerate + ATP
Phosphoglycerate mutase	I4Z8T9	3-phosphoglycerate ⇌ 2-phosphoglycerate
Enolase	I4ZCE6	2-phosphoglycerate ⇌ phosphoenolpyruvate + H_2O
Pyruvate kinase	I4ZA98	phosphoenolpyruvate + ADP → pyruvate + ATP
F_1F_O ATP synthase (proton-dependent)	I47BC8+9 I4ZBD1-6	ATP + (3-4) H^+_{in} ⇌ ADP + P_i + (3-4) H^+_{out}
Pyrophosphatase	I4Z7F1	PP_i + H_2O + n Na^+_{in} / n H^+_{in} ⇌ 2 P_i + n Na^+_{out} / n H^+_{out}
PEP carboxykinase	I4Z866	phosphoenolpyruvate + NDP + P_i + CO_2 ⇌ oxaloacetate + NTP
Malate dehydrogenase	I4Z8Y0	oxaloacetate + NADH ⇌ malate + NAD^+
Fumarase	I4ZA21	malate ⇌ fumarate + H_2O
Pyruvate oxidoreductase	I4ZAL7	Pyruvate + CoA + X_{ox} ⇌ acetyl-CoA + CO_2 + X_{red}
Phosphate acetyltransferase	I4Z807	acetyl-CoA + phosphate ⇌ acetyl phosphate + CoA
Acetate kinase	I4Z808	acetyl phosphate + ADP + P_i ⇌ acetate + ATP
AMP-forming acetyl-CoA synthase	A0A137T148	acetate + ATP + CoA → acetyl-CoA + AMP + PP_i
Quinol:fumarate oxidoreductase	I4Z8D8+9 I4Z8E0	QH_2 + fumarate ⇌ Q + succinate

Na^+ -translocating NADH:quinone oxidoreductase	I4ZAR9<br>I4ZAS0-4	NADH +H^+ + Q + 2 Na^+_{in} → NAD^+ + QH_2 + 2 Na^+_{out}
11-subunit complex (related to Nuo complex)	I4ZA64-73	unknown
NADH dehydrogenase 2 (non-electrogenic)	I4Z8L8	NADH +H^+ + Q → NAD^+ + QH_2
Cytochrome *bd* quinol oxidase	I4Z6X4-5	QH_2 + ½ O_2 → Q + H_2O
NhaA	I4ZAA4	Na^+_{in} + 2 H^+_{out} ⇌ Na^+_{out} + 2 H^+_{in}
NhaD	I4Z742	n Na^+_{in} + m H^+_{out} ⇌ n Na^+_{out} + m H^+_{in}<br>n Li^+_{in} + m H^+_{out} ⇌ n Li^+_{out} + m H^+_{in}

Table S2. Flavinylated subunits of the NQR and QFR in *P. bivia* membranes solubilized with 2.5 % DDM. Fluorescent bands indicating flavinylated proteins were excised from the SDS gel (see corresponding boxes highlighted in fig. 6), subjected to proteolysis, and analyzed by mass spectrometry. Total number of identified peptides and peptide sequences of FrdA, NqrB and NqrC are presented.

Box	Protein	Peptide number	Peptide sequence
1	FrdA	31	IPEGPVAEK; IPEGPVAEKWTNYK; RAHSIAAQGGINAAK; AHSIAAQGGINAAK; NYQNDGDSIYR; LAEVSNDIIDQCVAQGVPFAR; EYGGMLANR; SFGGAQVSR; GQTGQQLLLGAYSSLSAQVQTGK; LYTRYEMEDVVIVNGAAR; YEMEDVVIVNGAAR; NLITGKLER; LTLMSESLR; QGSDIPEEDRDYYLER; RYPAFGNLVPR; YPAFGNLVPR; GFGVNNTGLAVFLDFSESIER; LGLDVVLQR; YGNLFDMYEEITDVNPGEVANEINGVK; GLFAIGECNFSDHGANR; LGASALMQGLADGYFVLPYTIQNYLADQALWGHLSTDLPEFEEAEK; ELGHIMWEHVGMGR; TAEGLREGLEK; KEFDTNLFVPGTAEGLNVELDK; EFDTNLFVPGTAEGLNVELDK; DFILMGELVANDALSR; EEYQTEEGEAK; EEYQTEEGEAKR; ENLEYEAIK; ENLEYEAIKVQTR
2	NqrB	4	SVFDGFDTFLYVPNETSK; SGVSIHDAIDSKR; SGVSIHDAIDSK; AFLFFAYPSR
	NqrC	10	QILFALNQDR; QILFALNQDRDMTNPQAEK; DMTNPQAEK; EIITADDIINADGQVTTSGK; QGGIEAGFK; TVFGAYFNHESETAGLGAEIKDNK; KLFAAGDEK; LFAAGDEK; KIALSVMK; GSLQPYVK
